# The implications of population growth and climate change on sustainable development in Bangladesh

**DOI:** 10.4102/jamba.v11i1.535

**Published:** 2019-01-10

**Authors:** Md Sanaul H. Mondal

**Affiliations:** 1 Department of Transdisciplinary Science and Engineering, Tokyo Institute of Technology, Japan; 2 Department of Social Relations, East West University, Bangladesh

## Abstract

Bangladesh is characterised by its large population on a small land, rapid and unplanned urbanisation, rising urban inequalities, food and nutritional insecurity and lower level of resilience to climate change. These combined effects are major threats to food security of the country in the near future. This paper examined the implications of population growth and climate change on sustainable development of Bangladesh. This research was based on the analysis of chronological data and synthesis of literature on population growth, greenhouse gases emission, climate change, food security and sustainable development, mainly contextualised on Bangladesh. The analysis found that the population of Bangladesh has almost doubled between 1980 and 2015. The country shared around 2.2% (in 2013) of global population and contributed only 0.19% of global carbon dioxide emission. On the contrary, climate change is the biggest challenge for the country. An increase in temperature could decline rice and wheat production. Moreover, average monsoon rainfall would be increased as a result of increased temperature. The increase in temperature and rainfall may lead to early arrival and late departure of the monsoon season or an increase in mean daily rainfall intensity. Population growth and climate change have multiple implications on development. Therefore, sustainable development may be difficult to attain if climate change continues to jeopardise economic growth, environmental stability as well as the social progress of Bangladesh.

## Introduction

Bangladesh’s population is growing rapidly and will pass 200 million by 2045 (medium variant projection) (United Nations Department of Economic and Social Affairs Population Division [UNDESA] 2015). The population of Bangladesh has almost doubled over the last 35 years, to about 160.99 million people in 2015 from 81.36 million in 1980 (World Bank 2016). Population growth results in an increase in the absolute number of the population and an increase in the standard of living. These two determinants are associated with extraction and consumption of natural resources. The emission of greenhouse gases (GHGs) is a function of total population because every mouth has to be fed. The growing population is putting stress on agricultural production systems that aim to secure food production (Vetter et al. 2017). On the contrary, food production contributes a substantial amount of GHGs, including carbon dioxide (CO_2_), methane (CH_4_) and nitrous oxide to the atmosphere (Cohen 2010; Pitesky et al. 2009; Smith & Martino 2007; Steinfeld et al. 2006; Vetter et al. 2017; Wolf et al. 2010). Agriculture has a noteworthy contribution to ensure national food security, especially for developing countries like Bangladesh. Methane generated from agricultural practices is the second major source of GHGs emission in the world (United States Environmental Protection Agency [USEPA] 2018a). Furthermore, industrialisation and development interventions contribute enormous GHGs emissions (He 2014). GHGs are the most important driver of observed climate change on Earth since the mid-20th century (USEPA 2018b). In sober fact, the more population on Earth indicates more consumption and more emissions, which intensifies climate change.

Climate change is the single most pressing environmental issue for the Earth’s biotic environment with adverse implications for food security, freshwater supply and human health (United Nations Framework Convention on Climate Change [UNFCCC] 2007). Climate change is also the biggest challenge for tropical and subtropical countries of the world, especially for coastal areas and islands. The impact would be particularly severe in the tropical areas, which mainly consist of developing countries (Sathaye, Shukla & Ravindranath 2006), including Bangladesh. Bangladesh is exposed to climate-induced extreme events as a result of its geographical location (at the confluence of Ganges–Brahmaputra–Meghna [GBM] basin, Himalaya in the north and Bay of Bengal in the south), extremely low and flat terrain (almost 80% of total land area is floodplains and half of the area is within 10 m above the mean sea level), high population pressure (159.1 million in 2015) (World Bank 2016) and density (1237 people per km^2^ in 2015) (World Bank 2016), high levels of poverty, nature-dependent economic activities (42.9% people employed in agriculture sectors and comprising about 15.5% of gross domestic product (GDP) at current prices in 2015) (World Bank 2016), and so on. The above physical, social and economic conditions make the country the sixth most climate-affected countries of the world for the period of 1997–2016 (Eckstein, Künzel & Schäfer 2017). Therefore, climate change may attenuate the ability of the country to achieve sustainable development through weakening the sustainability of livelihoods, especially natural-based rural livelihoods and wipe-out development efforts.

Population includes the number of people; their demographic characteristics like age, sex, health, education and familial status; their demographic processes like birth, death, migration, the formation of unions and families and their dissolution; and the spatial distribution of people by geographic regions and size of settlements, from rural to urban (Cohen 2010). Therefore, population growth has diversified effects on development. On the contrary, the relationship between climate change and development is reciprocal. Social and economic development may be influenced by climate change, while society’s precedence on sustainable development influences the level of emissions of GHGs that are causing climate change (IPCC 2007). Sustainable development is defined by the Brundtland Report as ‘the development that meets the need and demand of the present generations without compromising the ability of future generations to meet their own needs’ (Brundtland 1987). The core concept of sustainability integrates economic, social and environmental aspect. Therefore, sustainable development may be difficult to attain if climate change continues to jeopardise economic growth, environmental stability as well as social progress. In reality, no country or nation is immune from the adverse impact of climate change. To realise present and future development gains, the Sustainable Development Goals (SDGs) have incorporated ‘(tackling) climate change’ as one of the important pillars to sustain the development beyond 2030 (United Nations 2018). Being a densely populated and developing country, Bangladesh is particularly vulnerable to climate change and will have the least options to cope with the projected climatic shocks.

Much research has been conducted on the impact of population growth and/or climate change on sustainable development at global scale (e.g. African Institute for Development Policy [AFIDEP] & Population Action International [PAI] 2012; Bremner et al. 2010; Cohen 2010; Donner & Rodríguez 2008; Jiang & Hardee 2011; Schneider et al. 2011; Sherbinin et al. 2007); however, limited research has so far been conducted on those aspects in Bangladesh. The study of Islam and Islam (2017) focused on impact of climate change on sustainable development of the country. The demand of rice and wheat for the year 2050 with the projected population scenarios was calculated by Mainuddin and Kirby (2015) to analyse the food security status of Bangladesh. In their paper, Faisal and Parveen (2004) studied the implication of food security of the country in consideration with climate change, population growth and limited resources. Solaiman and Belal (1999) proposed a sustainable development process framework of the country which sets four interrelated issues: stabilisation of population, food security, optimum uses of natural resources and growth of economy and industry. Mahtab and Karim (1992) set forth an analysis of agricultural carrying capacity with the projected population growth and land-use changes. All these studies, however, focused on either population or climate change. Thus, this study was an effort to investigate the implications of population growth and climate change of Bangladesh from a sustainability lens.

### Conceptual framework

**FIGURE 1:**
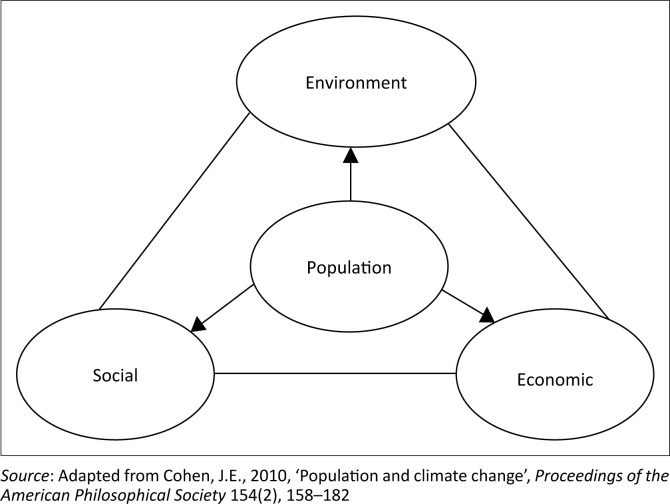
Interactions between human population and sustainable development.

The connections between population and environment (climate factors) depend on social (cultural factors) and economic aspects. These tetrahedron relationships can be visualised with a triangular base and a point at the centre of the triangle (Figure 1). The vertex of the triangle refers to the environment. The other two corners of the triangle indicate social and economic dimensions (these are the three pillars of sustainable development). Population is placed at the centre of the triangle. This figure is a graphical representation of how the population is interrelated and affected by environmental, social and economic parameters. For example, a society with a small population size and higher economic growth (e.g. developed countries) may generate more emission than a society with a large population and small economy (e.g. developing countries), and vice versa. Technology (social or cultural) can also reduce emission pattern even with big population size and large economy. This study explored the relationship between population growth, climate change and sustainable development of Bangladesh using the following framework as shown in Figure 1.

### Objectives of the research

Bangladesh is characterised by rapid population growth, high density, unplanned urbanisation, increasing urban inequalities, food and nutritional insecurity, as well as lower level of resilience to the impact of climate change. The combined effects of population growth and climate change are major threats to food security for the country. Taking all these factors into consideration, this study aims at examining the implications of population growth and climate change on sustainable development of Bangladesh. To analyse these multidisciplinary aspects, this paper first establishes the correlation among population growth, GDP growth and CO_2_ emission of Bangladesh. This is followed by a synthesis of the social, economic and environmental implications of population growth and climate change on sustainable development of the country. The results of this study would help policymakers and development practitioners to deal with population growth and climate change as these coincide with development agenda for sustainable development.

## Methods and materials

**FIGURE 2:**
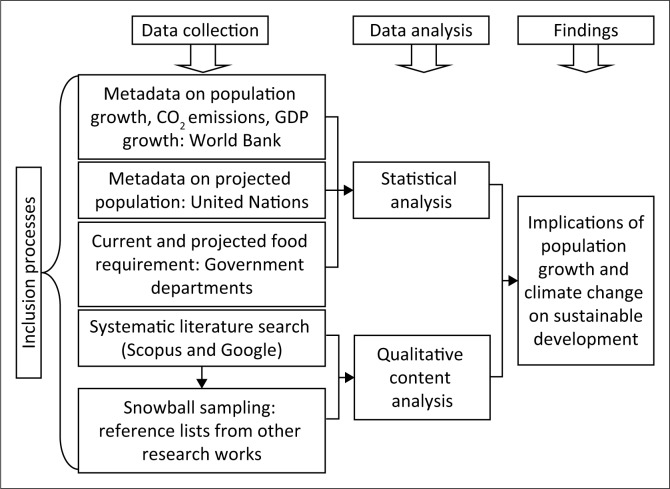
Methodological flow chart of this study.

This research used exploratory research design. Data for this study were gathered from various sources (Figure 2). Scopus and Google search engines were used to collect relevant literature on population growth, GHG emission, climate change, food security and sustainable development of Bangladesh using keywords (population, climate change, sustainable development and Bangladesh). Snowball sampling is a non-probability sampling method that engages data sources recommended by other possible data sources to be used in a research (Mugambiwa & Tirivangasi 2017). Snowball sampling was also employed for this study.

The data on population growth, CO_2_ emissions and GDP were collected from World Bank (2016) for the period 1972–2013. The data were then used to derivate the correlation between population size and CO_2_ emissions; population size and GDP; and CO_2_ emissions and GDP applying Pearson correlation coefficient (Equation 1). The calculations and output were made possible using Statistical Package for the Social Sciences version 17 software.

Calculation of Pearson correlation coefficient:

$$r=\frac{\sum_{i}\left(x_{i}-\bar{x})(y_{i}-\bar{y}\right)}{\sqrt{\sum_{i}{\left(x_{i}-\bar{x}\right)}^{2}\sqrt{\sum_{i}{\left(y_{i}-\bar{y}\right)}^{2}}}}$$ [Eqn 1]

Projected population growth and urbanisation rate of Bangladesh for different periods were estimated from the United Nations Department of Economic and Social Affairs Population Division (UNDESA). Current food production (e.g. rice, wheat, maize, pulses, oil-seed and vegetable) for the period 2015–2016 and projected food requirement by 2030 were derived from the government sources Bangladesh Bureau of Statistics and Bangladesh Agriculture Research Council, respectively. Those data sources were chosen based on the availability of required data from a single source.

## Results and discussions

### Growth trend of population, gross domestic product and carbon dioxide emissions

The annual rate of population growth and GDP growth for Bangladesh in 2013 was 1.22% and 6.01%, respectively, whereas annual growth rate of CO_2_ emission was about 2.13% in 2013 (Table 1) (World Bank 2016). More specifically, each 1% increase in population in 2013 was associated with a 1.75% increase in CO_2_ emissions, whereas each 1% increase in annual GDP was associated with a 0.35% increase in CO_2_ emissions (World Bank 2016).

**TABLE 1:** Growth of population, gross domestic product and carbon dioxide in Bangladesh.

Parameter	Year	
	1992	2013
Total population	110 987 459	157 157 394
Population growth (annual %)	2.26	1.22
GDP (current US$, in millions)	31 708.87	149 990.45
GDP growth (annual %)	5.44	6.01
GDP per capita (current US$)	285.69	954.39
GDP per capita growth (annual %)	3.09	4.72
CO_2_ emissions (kt)	17 748	68.510
CO_2_ emissions (metric tons per capita)	0.16	0.44

*Source*: Adapted from World Bank, 2016, *World development indicators*, viewed 28 October 2016, from http://data.worldbank.org/indicator

GDP, gross domestic product; kt, kilo tons.

During the 20-year period between 1992 and 2013, the population of Bangladesh grew by a factor of 1.42, GDP per capita increased 3.34 fold and CO_2_ emission per capita increased 2.74 fold (Table 1). Here, one may easily conclude population growth contributed more CO_2_ emission than GDP growth. But there continues to be a debate about whether population growth or increasing consumption levels have contributed relatively more to GHGs emission (Dietz, Rosa & York 2007; Jiang & Hardee 2011; Meyerson 1998; Parikh & Painuly 1994). The economy of the country is running through a process of transition (Solaiman & Belal 1999). In reality, GHG emissions per person depend on income, technology, demographic factors, institutional and economic factors, and a host of behavioural factors (Cohen 2010). In this regard, the result of Pearson correlation analysis on time series data (from 1972 to 2012) of population growth, GDP growth and GHGs emission was different. Results obtained from the Pearson correlation analysis (Table 2) presented three bivariate relationships. In all cases, positive correlations observed were statistically significant (i.e. population size and CO_2_ emissions were positively correlated; population size and GDP were positively correlated; and CO_2_ emissions and GDP were positively correlated), meaning that those variables tend to increase together (i.e. increasing GDP associated with increasing CO_2_ emission). Although the three bivariate Pearson correlation (1) population size and CO_2_ emissions (*r* = 0.928; *p* < 0.01), (2) population size and GDP (*r* = 0.907; *p* < 0.01), and (3) GDP and CO_2_ emissions (*r* = 0.987; *p* < 0.01) showed very strong and positive correlations, the correlation coefficient values were not the same. The correlation coefficient between CO_2_ emissions and GDP was very strong compared with the other two bivariate models. This analysis identified that increase in GDP growth depends positively on the emission of GHGs. Therefore, the relationship between the CO_2_ emission and economic growth has a significant implication for the environment and economy (Deviren & Deviren 2016) of Bangladesh, as the country has put more focus on its economic development in recent decades.

**TABLE 2:** Correlation analysis of among population size, carbon dioxide emissions size and gross domestic product (from 1972 to 2012).

Variables	Population size	GDP (current US$)	Total GHG emissions (kt of CO_2_ equivalent)
Population size			
Pearson correlation	1.000	0.907†	0.928†
Sig. (1-tailed)	-	0	0
*N*	41	41	41
**GDP (current US$)**			
Pearson correlation	0.907†	1.000	0.987†
Sig. (1-tailed)	-	0	0
*N*	41	41	41
**Total GHG emissions (kt of CO_2_ equivalent)**			
Pearson correlation	0.928†	0.987†	1.000
Sig. (1-tailed)	-	0	-
*N*	41	41	41

*Source*: Author’s calculation adapting the time series data from World Bank, 2016, World development indicators, viewed 28 October 2016, from http://data.worldbank.org/indicator

† , Correlation is significant at the 0.01 level (1-tailed).

Sig., significance; GDP, gross domestic product; kt, kilo tons; US$, US dollar; GHG, greenhouse gas; CO_2_, carbon dioxide.

### Implications of population growth on sustainable development in Bangladesh

**FIGURE 3:**
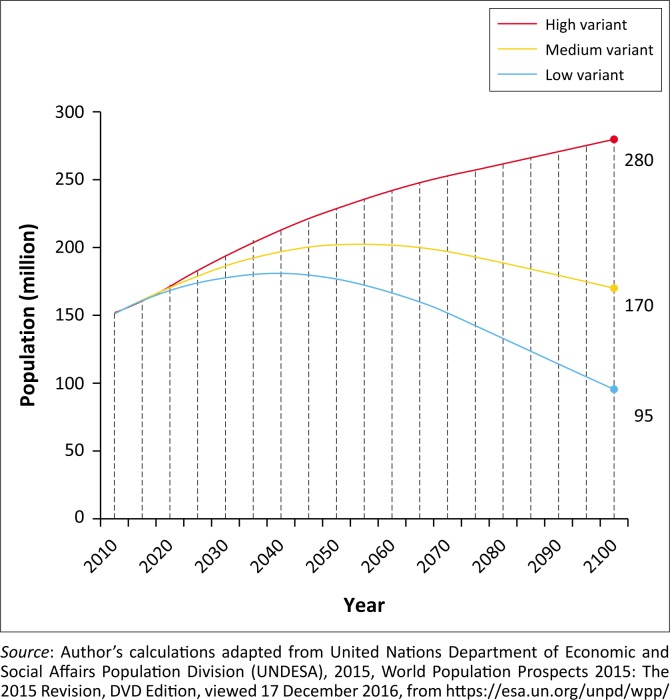
Population of Bangladesh (based on high, medium and low projection of the United Nations).

The population of Bangladesh increased from around 69.84 million in 1974 to about 153.41 million in 2011 (World Bank 2016) and will reach 201.64 million by 2061 (Figure 3) (medium variant projection) (UNDESA 2015). This growth of population was largely accompanied by the high rate of total fertility (total number of children per woman) in the past few decades. The total fertility rate (TFR) was 6.8 children per woman in 1975 and decreased to 2.2 children per woman in 2014 (World Bank 2016). According to the United Nations (medium variant projection), TFR of Bangladesh will further decline to 1.95 children per woman by 2020 (UNDESA 2015). This decline in fertility was attributed to increased use of modern contraception methods, which increased from 7.7% in 1975 (Ministry of Health and Population Control [MOHPC] 1978) to 62.4% in 2014 (National Institute of Population Research and Training [NIPORT], Mitra and Associates & ICF International 2016). In 2014, unmet need for family planning (those who wanted to postpone their next child or stop childbearing but not currently practicing contraception) was 12% (NIPORT, Mitra and Associates & ICF International 2016), which was a decline from 21.6% in 1993–94 (NIPORT, Mitra and Associates & ICF International 1994). In addition, infant mortality rate decreased from 74 per 1000 live births in 1970 to 38 per 1000 live births in 2010 (NIPORT, Mitra and Associates & ICF International 2016).

This shift in fertility and mortality profiles has resulted in a significant transition in the age structure of the population. This is characterised by an increase in the working-age population (15–59 years). In 2015, around 65.6% of Bangladesh’s population was dominated by the working-age group people (World Bank 2016). The age-structural transition (more people in the working-age group) creates a demographic window of opportunity or demographic dividend for economic growth resulting from greater savings generation and increased productivity as a result of the smaller number of dependents. However, benefits from demographic dividend are optimised when accompanied by investments in health and education, pro-growth and job-creating economic reforms (AFIDEP & PAI 2012).

**FIGURE 4:**
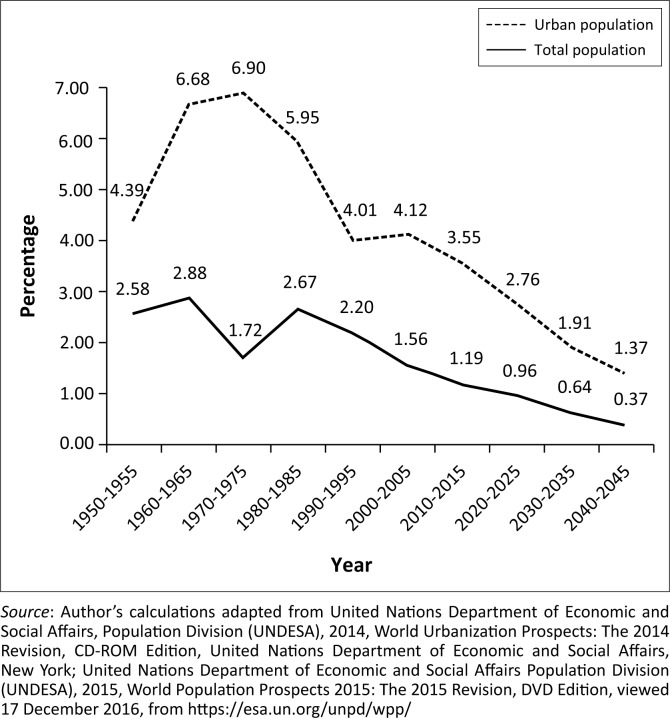
Average annual rate of change of total population and urbanisation (in per cent).

Urbanisation has brought significant positive changes in Bangladesh, even though it has been a huge challenge environmentally, socially and economically (Rana 2011). Most of the urban centres of the country are experiencing rapid population growth and unplanned urbanisation and migration. Historical and projected data revealed that the average annual rate of changes in urban population is always higher compared to changes in the annual rate of total population in Bangladesh as shown in Figure 4.

Urban population in Bangladesh reached 55.2 million in 2015 (34.3% of total population) (World Bank 2016) and is likely to reach 83.2 million (45% of total population) in 2030 and 112.4 million (56% of total population) in 2050 (UNDESA 2014). This rapid growth of urban population will add further strains on urban basic services such as water supply, sanitation, drainage management and even on transportation services. Urban areas of the country are already facing the challenges of accommodating the burgeoning population. As a result, most of the urban dwellers live in slums or informal settlements. The slum dwellers increased from around 18.4 million (87.3% of the total urban population) in 1990 to 29.4 million (55.1% of the total urban population) in 2014 (World Bank 2016). All the health outcomes for slum dwellers are poor as they live in poor housing; lack access to health facilities, pure drinking water, and sanitation; as well as poor environmental conditions. Therefore, the growth of urban poverty is now a serious development concern for the country. Climate change further multiplies the challenges associated with the rapid growth of urban population by increasing vulnerabilities of the urban dwellers, particularly for those cities located in low-lying coastal zones that are prone to sea-level rise (AFIDEP & PAI 2012). On the contrary, slum dwellers are highly susceptible to climate-related abnormalities such as flooding. Around 8% of urban land areas have an elevation of less than 5 million where 1.8% of country’s total population was living in 2010 (World Bank 2016). An increase in urban population is also associated with the horizontal expansion of urban area and encroachment of productive farmlands. The environmental stress of urban population is multiplied by the luxurious consumption patterns and use of technology (Solaiman & Belal 1999). Population stabilisation in both rural and urban areas is, therefore, a crucial issue for social, economic and environmental sustainability of the country.

The human health risk is one of the potential emerging risks from climate change of the country (Kabir et al. 2016). The working-age population (15–59 years) of the country is projected (medium variant projection) to be around 65.46% by 2030 with 11.5% ageing population (60+ years) (UNDESA 2015). Changes in the demographic structure must pose a serious disease burden on the country, such as, extreme temperatures could contribute to deaths from cardiovascular or respiratory diseases, frequent cyclones may lead to water-borne diseases and displacement of population, and so on. Climate change will also increase the risk of communicable diseases. If public health systems are inadequate or if new pathogens or vectors arise that are resistant to the country’s current methods of disease control, it may lead to declining life expectancies and diminishing economic productivity (Rahman 2008). Consequently, population dynamics is very important for sustainable development of the country.

### Implications of climate change on sustainable development in Bangladesh

#### Greenhouse gases emission dilemma in Bangladesh

The Intergovernmental Panel on Climate Change (IPCC) (First Assessment Report in 1990) projected that the global mean temperature could increase at an average rate of 0.3 °C per decade with a ‘business-as-usual’ emissions scenario of GHGs and could reach 3.3 °C by the year 2100 (within a range of uncertainty of 2.2 °C – 4.9 °C) (IPCC 1990). The concentration of GHGs in the Earth’s atmosphere has been increasing after the industrial revolution. The concentration of CO_2_ in the atmosphere in the pre-industrial period was 280 parts per million by volume (ppmv) and rose to over 350 ppmv by 1990 (Warrick, Bhuiya & Mirza 1993). The estimated concentration of CO_2_ in 2016 heaved over 404.07 ppmv (Dlugokencky & Tans 2017). Over the last three decades (from 1986 to 2016), the concentration of CO_2_ has been increasing at a rate of around 0.53% per annum (Dlugokencky & Tans 2017). During 1750–2016, the atmospheric concentration of CO_2_ has increased by 44%.

It is quite evident that the largest share of GHGs emission in the atmosphere (historical and current global emission of GHGs) has been contributed by the developed countries. In 2013, United States and Bangladesh shared 4.4% and 2.2% of global population and contributed 14.5% and 0.19% of global CO_2_ emissions, respectively (World Bank 2016). In the United States, average emission of CO_2_ per person per year was over 16.4 metric tons in 2013 (World Bank 2016). Whereas, per capita emissions of CO_2_ for a Bangladeshi was 0.44 metric tons per year in the same year (World Bank 2016), which was around 1/37 the amount of an average American. Furthermore, on a global scale, the per capita emission of CO_2_ for the same year was 4.99 metric tons (World Bank 2016). These statistics clearly depict the geographical disparities of CO_2_ emission. In contrast, the emitted CO_2_ is rapidly and evenly distributed around the globe. Methane is another GHG, which has been increasing as a result of human activities like agriculture (rice production, animal husbandry), solid waste disposal, fossil fuel–related leakage associated with gas pipelines and coal mining, landfills and biomass burning. Bangladesh makes a contribution (albeit a minor one) to the yearly emission of methane because of its extensive areas of seasonally or perennially flooded land and wetland paddy cultivation, as well as its animal husbandry (Warrick et al. 1993). Thus, increasing concentration of GHGs should result in global warming and future climate change. In summary, the average contribution of GHGs emission from Bangladeshis is far less from Americans or even the global average. In a nutshell, increasing concentration of GHGs in the atmosphere should cause the world to warm. However, there is an uncertainty of the rate of warming. Whatever the contribution of GHGs emission and concentration of GHGs in the atmosphere of Bangladesh, climate change (because of global emission and other anthropogenic interventions) must pose a serious threat to its environmental, social and economic systems. The vicious impacts of climate change for Bangladesh would be increased flood damage to infrastructure, livelihoods and settlements; increased heat-related human mortality; and increased drought related to water and food shortage.

#### How does climate change matter for sustainable development of Bangladesh?

**FIGURE 5:**
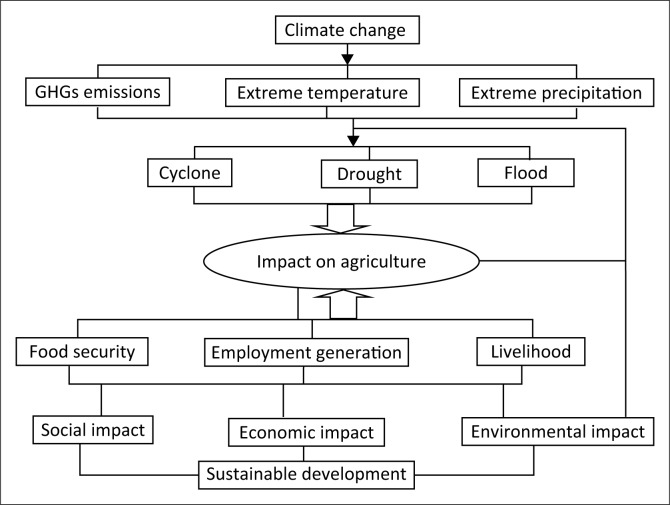
Conceptualisation of implication of climate change on sustainable development (greenhouse gas).

The shock of climate change on the globe is unevenly distributed. The awful impacts will face by developing countries because of their geography; poor coping capacities; and vulnerable social, institutional and physical infrastructures (Banuri & Opschoor 2007). Bangladesh, as a developing country, has undergone from conventional risk to new systematic risk like climatic change. This risk transition is very crucial for Bangladesh because climate change will increase the risk of extreme climatic events like floods, droughts and tropical cyclones. This risk transition in the form of climate change is very crucial for Bangladesh, as its economy is still pillared on climate-sensitive primary economic activities like agriculture. Food production is expected to be severely disrupted by frequent flooding in the rainy season and by drought in dry season as a result of the scarcity of water. The widespread flood in 1987, 1988, 1998 and 2004 resulted in 3927 deaths (Emergency Events Database [EM-DAT] 2016); drought of 1989 affected around 47% of land and 53% of population; and super cyclone of 1991, 2007 (Sidr) and 2009 (Aila) resulted in 143 338 deaths (EM-DAT 2016) are the very recent reminder of the degree to which people of Bangladesh are susceptible to the changes in climatic variability. The 1987 flood alone caused 2055 deaths (EM-DAT 2016), inundated over 50 000 km^2^ and caused damage approximating $1.0 billion (Ahmed & Suphachalasai 2014). The estimated damage by cyclone Sidr and cyclone Aila was around $1.7 billion and $270 million, respectively (World Bank 2016). Drought is a slow-onset climatological event for Bangladesh (Tanner et al. 2007). Very severe droughts hit the country in 1951, 1957, 1958, 1961, 1972, 1975, 1979, 1981, 1982, 1984 and 1989 (Ahmed & Suphachalasai 2014). For example, the drought of 1978 and 1979 affected around 42% of the cultivable land with a decrease of around 2 million tons (MT) rice production (Brammer 1987). Climate change will also influence basic human rights, including the rights to life, housing, food, water, health, adequate standard of living, and culture, education, and self-determination (Lofts et al. 2017). Therefore, the socio-economic and environmental cost of cyclone, drought and flood would be higher because of increased population in its floodplains. In this section, the implications of climate change on sustainable development are conceptualised (Figure 5) by the impacts on only the agricultural sector on which the country’s food security and economic activities largely rely.

**Implications on environment:** Climate change induced by global warming could increase melting of glaciers in the Himalayas and thus the normal hydrological cycle may be disturbed. Disruption in hydrological cycle may alter frequency and severity of cyclonic storms and floods in the coastal zone and droughts and floods in the northern zone of the country.

**GHGs emission:** Agriculture is extremely influenced by climate (factors), which in turn is affected by agricultural activities through emitting GHGs. Increased concentration of CO_2_ in the atmosphere could increase photosynthesis rate and efficiency of water use by plants, which may increase the growth and yield of plants (Warrick et al. 1993). Rice, wheat, jute and pulses would benefit by increased CO_2_. However, increased level of CO_2_, temperature and precipitation could favour insect, pests and plants diseases. Furthermore, crop production will further decline by reduced intensity of light, increased frequency of floods and cyclones and salinity ingression by surges in the coastal zone (Warrick et al. 1993). With a moderate climate change scenario, the estimated crop loss because of salinity ingression in the coastal zone may be around 0.2 MT (Habibullah, Ahmed & Karim 1998).

**Extreme temperature:** The projected temperature increases in Bangladesh for the three periods are: 0.9 °C – 1.9°C, 1.6 °C – 2.5 °C and 2.9 °C – 4.2 °C by 2030, 2050 and 2080, respectively (Ahmed & Suphachalasai 2014). Such increase in temperature would have both positive and negative outcomes in terms of early arrival and delayed departure of monsoon season or increase in average daily rainfall receives. Moreover, higher temperatures would increase evaporation, which may lead to drought in dry season (winter season). This could increase greater dependence on groundwater extraction. In addition, increased temperatures because of global warming could adversely affect dry season crop production through shortening the winter season. In reality, higher temperatures are beneficial for *Aus* rice (rain-fed rice), *Aman* rice (rain-fed rice) and jute (monsoon crops), whereas, the yield of *Boro* rice (irrigated rice), wheat, potato and pulses (winter or dry season crops) will be reduced because of increased winter temperatures. An increase in 4 °C temperature could decline around 28% of rice production and 68% of wheat production (Ahmed & Suphachalasai 2014). Moreover, *Boro* rice yield may be declined by 1.5% by 2020, 2.5% by 2030, 4.4% by 2040 and 5.4% by 2050 under projected climate change scenario (IPCC 2007). Therefore, the relation between climatic variability and agriculture is very crucial for the country because climatic factors strongly interact with crop yield.

**Extreme precipitation:** Agriculture is vulnerable to moisture deficiency, prolonged inundation, excessive rainfall, flood, cyclone, salinity, and so on. Climate change could alter the spatial and temporal distribution and frequency, intensity and duration of natural disasters and its types. Most of the climate models for Bangladesh showed an increase in mean annual rainfall. The ‘best estimate’ scenario for 2030 is that monsoon rainfall could increase by 10% to 15% and winter rainfall by 5% to 10% (Ahmad et al. 1994). Increased rainfall could reduce the frequency of drought, lessen irrigation demand, increase recharge of groundwater and increase the flow of rivers. The average water flow of Ganges-Brahmaputra-Meghna (GBM) River may increase by 6%, 15% and 19%, respectively, with a rise of global mean temperature by 2 °C (IPCC 2001; Mirza et al. 1998; Rasheed 2016). In general, increased rainfall in GBM basin could increase the frequency and severity of floods in Bangladesh, which may outweigh the positive impacts of climate change because of the severity of damage by floods. In general, moisture content in soil may be increased because of increased rainfall, which would be beneficial for dry season crops. On the other hand, excessive moisture in the soil could reduce the water-holding capacity of the soil, which may result in waterlogged conditions. Prolonged waterlogging coupled with poor drainage would destroy dry season (*Rabi* season) cultivation. Moreover, climate-induced decreased precipitation in winter (dry) season could increase the risk for drought risk demand for irrigation in the *Rabi* season (dry season). Furthermore, increased rainfall in the tertiary period hilly area (Chittagong hill tracts) may speed up top soil erosion and thus, the entire hill forest will become vulnerable to climate change. Although climate change–induced additional flooding would supply more water to the forests, the soils of *Sal* (*Shorea Robusta*) forest in *Madhupur* and *Barind* tracts, which have low moisture retention capacity in winter, would face additional moisture stress and moderate drought conditions because of higher evaporation rates (World Bank 2000).

**Socio-economic implications:** Agriculture is the most valuable economic sector of the country because of its triple roles: ensuring food security, generating employment and providing livelihoods for around half of the labour force of the country. Although the country has made significant agricultural development in recent decades, the emerging impacts of climate change may pose serious threats to food security of the people, especially the poor and marginalised (Habiba et al. 2015).

**Implication on food security:** Climatic condition affects pattern and production rate of different crops (FAO 2011). Therefore, food security is a major concern for Bangladesh under projected climate change. Climate change will affect directly (crop yield) and indirectly (access to food and availability of food) food security. Although Bangladesh achieves remarkable progress in domestic food production, the majority of the population faces challenges accessing a diet of adequate quantity and diversity necessary for a healthy and productive life (World Food Programme [WFP] 2015). Therefore, ensuring sustainable food security is essential. The major challenges of sustainable food security include increase in population size, decrease in nutritional security, financial inability to access to food, shrink agricultural land and strain on natural resources and human and plants health (Habiba et al. 2015; Jain, Hansra & Chakraborty 2010).

Poverty and food insecurity are interrelated to each other in many ways. The crop production of the country will be adversely affected by climate change (Timsina et al. 2018). Climate change may supress the target of achieving food security in Bangladesh, which further hampers the goals of 2030 global agenda through worsening achievement of ‘end poverty’ and zero hunger (UN 2018). In Bangladesh, food security is synonymous with the term ‘food self-sufficiency’ (Faisal & Parveen 2004). The country has achieved self-sufficiency in rice production in the recent years (Chen & Lu 2018; Timsina et al. 2018). The estimated food requirement by 2030 and present food production (2015–2016) of different crops have been indicated in Table 3. It is quite evident that the gap between these two periods for all crops will be enormously wide as per the projected requirement (National Agricultural Technology Project [NATP] 2012). This gap will further increase under climate change. For example, the flood of 2017 in northwestern and northeastern parts of the country has enormous implications on the impact of climate-induced disasters, which destroyed MT of rice resulting in price hike and imports of foods. Rice import in this fiscal year (2017–2018) is higher compared with the imports in the last three decades. The import of rice during the last two quarters of this fiscal year (July 2017 – December 2017) stood at 2.29 MT in contrast to 0.13 MT in the fiscal year 2016–2017 (Food Planning and Monitoring Unit [FPMU] 2017). Hence, ensuring food security under projected climate change to its large population would be challenging for the government.

**TABLE 3:** Current food production (in million ton) and projected food requirement by 2030.

Status	Year	Population (in millions)	Food production (in million tons)					
			Rice	Wheat	Maize	Pulses	Oil-seed	Vegetable
Current^†^	2015–2016	160.99	34.71	1.35	2.45	0.38	0.93	3.82
Projected^‡^	2030	186.46	39.80	3.85	4.00	3.50	1.70	13.98
Gap (%)		25.47	5.09	2.50	1.55	3.12	0.77	10.16

*Source*: Adapted from Bangladesh Bureau of Statistics (BBS), 2017, Year book of Agricultural Statistics-2016, Bangladesh Bureau of Statistics, Dhaka; National Agricultural Technology Project (NATP), 2012, Agricultural Research Vision 2030, Bangladesh Agricultural Development Corporation, Dhaka; United Nations Department of Economic and Social Affairs Population Division (UNDESA), 2015, World Population Prospects 2015: The 2015 Revision, DVD Edition, viewed 17 December 2016, from https://esa.un.org/unpd/wpp/; World Bank, 2016, World development indicators, viewed 28 October 2016, from http://data.worldbank.org/indicator

† , Population (in 2015): World Bank 2016, food production: BBS 2017;

‡ , Population: UNDESA 2015, food production: NATP 2012.

Because food security is directly influenced by climate, it is quite impossible to achieve the targets of SDGs without properly treating the issues related to climate change. For a country like Bangladesh that depends on rice for survival, a major loss of production could translate to a dire nutrition crisis. Thus, changes in climate variability could affect the sustainability of agricultural systems of the country, which will ultimately disrupt the production function and sustainable development.

**Implication on employment generation and providing livelihoods:** Although the share of agriculture to GDP decreased from 59.6% in 1972 to 15.5% in 2015 (World Bank 2016), still 47.5% labour force (World Bank 2016) of the country are engaged in this sector. Between 1990 and 2013, the arable lands of the country decreased by 19% (from 9.46 million ha in 1990 to 7.68 million hectares in 2013) and per capita arable lands decreased by 45% (from 0.089 ha/person in 1990 to 0.049 ha/person in 2013) (World Bank 2016). Nearly 65% (105.8 million) of the population of Bangladesh was living in rural areas in 2016 and more than half of them, directly and indirectly, depend on agriculture (World Bank 2016). Rural people mostly depend on climate-sensitive primary economic activities. Around 20% coastal land of the country will be submerged, if sea levels increas by 1 million and this situation may displace 25–30 million coastal populations (Agrawala & Berg 2002; Ahmed 2006; Faisal & Parveen 2004; Habiba et al. 2015; Habibullah et al. 1998; World Bank 2000). The Sundarbans mangrove forest ecosystem is also extremely vulnerable to climate-induced disasters in many ways. Changes in bio-physiography of coastal regions may have an adverse impact on Sundarbans, and lives and livelihoods of millions of frontier populations (Mondal 2017). This, inundation of coastal zone (including Sundarbans) might have at least three serious negative impacts: displacement of people, shrinking precious agricultural land and above all withdrawal of people from agriculture and other nature-dependent livelihoods. Such changes may leave the poor people in poverty and ultimately the target of ‘End Poverty’ (SDG 1) and ‘End Hunger’ (SDG 2) will be diminished (UN 2018). Hence, the outcome of climate change for Bangladesh is challenging because it could have negative effects on its agriculture on which the country’s economy largely depends.

## Limitations of the study

The availability and quality of recorded data in Bangladesh are very poor, and even the data for a particular variable might vary among different departments of government. The major challenge of this study was related to variations in metadata in different sources, and therefore, this paper did not claim itself as an unbiased study. A clear bias is observed for the selection of data from different sources. It is obvious that the data from World Bank or United Nations or other sources might be dissimilar from the data of government of Bangladesh. To overcome this limitation, the author considered similar data sources for a particular analysis, for example, retrospective data on population, CO_2_ emissions and GDP were retrieved from World Bank, prospective data on population growth and urbanisation were gathered from United Nations and rest of the data were collected from the government sources. To have a good track on SDG indicators, Bangladesh should strengthen its capacity to collect and report national statistics on an agreed set of criteria that comply with international standards (Sarvajayakesavalu 2015).

## Conclusion

Bangladesh is successful to reduce population growth rate, TFR, infant mortality rate and so on. The country is also a role model for developing countries because its GDP has more than tripled since 1971. Despite those socio-economic progresses, the country is at high risk of climate change which may undermine the social and economic stability of the country.

Bangladesh has already invested millions of dollars to adapt (structural adaptation and non-structural adaptation) with changing climate variability, for example, construction of cyclone shelters, strengthening the early-warning system, allocation of more budget in agriculture research, etc. Nevertheless, climate victims are not waiting for support from the top and have already started to cope with the adverse situation. But adaptation has its elastic limit. It is quite unclear, how much adaptive capacities of the communities can make the people climate proof? However, the cost of adaptation is somehow difficult to ascertain for Bangladesh. Indeed, various laboratory works estimate the cost of adaptation for Bangladesh; nevertheless, it might fall into a fallacy because ground reality for adaptation and mitigation efforts would be different from that of the laboratory.

Bangladesh is now self-sufficient to feed its large number of people at the cost of maximum intensification of agricultural lands. But the national priority to ensure food security might face severe challenge by the population growth, over cultivation, unplanned utilisation of lands and above all change in climate variability, and these factors might lead the country towards short-term or chronic food insecurity, which will hamper the development efforts of the country. The impact of 2017 flood is the reflection of the above assumptions. The question asked is: how best can the country prepare for climate change? This actually lies in population control, increasing access to quality education, raising income level through creating sustainable employment opportunities, enhancing community-level resilience to disasters, and above all ensuring good governance.

Bangladesh should take cohesive actions on climate change for eliminating extreme poverty and inequality and promoting sustainable development. Impacts of climate change in this country are not a future threat – they are already pervasive through increased frequency and intensity of tropical cyclones, increasing sea levels, decreasing crop yields and water shortages during dry seasons. Actions on climate change are very imperative, as climate change will pose threats to its achievement of SDGs. These actions must be integrated, balanced and inclusive, irrespective of social, economic, environmental and political arena to sustain the achievements of SDGs beyond 2030. To sum up, Bangladesh should prioritise population growth and climate change as an apex development agenda in the coming decades to achieve sustainable development.
